# *Chlamydia pneumoniae* Hides inside Apoptotic Neutrophils to Silently Infect and Propagate in Macrophages

**DOI:** 10.1371/journal.pone.0006020

**Published:** 2009-06-23

**Authors:** Jan Rupp, Lisa Pfleiderer, Christiane Jugert, Sonja Moeller, Matthias Klinger, Klaus Dalhoff, Werner Solbach, Steffen Stenger, Tamas Laskay, Ger van Zandbergen

**Affiliations:** 1 Institute of Medical Microbiology and Hygiene, University of Luebeck, Luebeck, Germany; 2 Medical Clinic III, University hospital Schleswig-Holstein, Luebeck, Germany; 3 Institute of Medical Microbiology and Hygiene, University Clinic of Ulm, Ulm, Germany; 4 Institute of Anatomy, University of Luebeck, Luebeck, Germany; University of California Merced, United States of America

## Abstract

**Background:**

Intracellular pathogens have developed elaborate strategies for silent infection of preferred host cells. *Chlamydia pneumoniae* is a common pathogen in acute infections of the respiratory tract (e.g. pneumonia) and associated with chronic lung sequelae in adults and children. Within the lung, alveolar macrophages and polymorph nuclear neutrophils (PMN) are the first line of defense against bacteria, but also preferred host phagocytes of chlamydiae.

**Methodology/Principal Findings:**

We could show that *C. pneumoniae* easily infect and hide inside neutrophil granulocytes until these cells become apoptotic and are subsequently taken up by macrophages. *C. pneumoniae* infection of macrophages via apoptotic PMN results in enhanced replicative activity of chlamydiae when compared to direct infection of macrophages, which results in persistence of the pathogen. Inhibition of the apoptotic recognition of *C. pneumoniae* infected PMN using PS- masking Annexin A5 significantly lowered the transmission of chlamydial infection to macrophages. Transfer of apoptotic *C. pneumoniae* infected PMN to macrophages resulted in an increased TGF-ß production, whereas direct infection of macrophages with chlamydiae was characterized by an enhanced TNF-α response.

**Conclusions/Significance:**

Taken together, our data suggest that *C. pneumoniae* uses neutrophil granulocytes to be silently taken up by long-lived macrophages, which allows for efficient propagation and immune protection within the human host.

## Introduction

*Chlamydia pneumoniae* is an obligate intracellular pathogen that enters the human body after respiratory infection. Infection activates airway epithelial cells resulting in a rapid recruitment of polymorph nuclear neutrophil granulocytes (PMN) [1], [2]. Consequently, in the lung, PMN are among the first leukocytes to encounter *C. pneumoniae* [3]. Importantly, phagocytosed *C. pneumoniae* are not killed; the ingested bacteria survive and multiply within PMN [4]. During the later course of the infection, viable chlamydiae are found inside alveolar macrophages (AM), bronchial/alveolar epithelial cells, vascular endothelial/smooth muscle cells and monocyte- derived macrophages (MF) [4]–[8].

Chlamydiae undergo a biphasic developmental cycle inside an internalized vesicle termed inclusion. Non dividing elementary bodies (EBs) change into the dividing and metabolically active reticulate bodies (RBs), re-differentiate to EBs and finally escape from the host cell [9]. Productive infection and chlamydial growth can be analyzed by inclusion morphology and size, but also by monitoring transcriptional activity of chlamydial genes involved in pathogen metabolism and pathogenicity throughout the developmental cycle [10]–[12]. Increased production of the pro-inflammatory cytokines IL-1ß and TNF-α have been demonstrated as markers of direct host immune responses to chlamydial infections in phagocytes [13], [14], which may result in intracellular killing of the bacteria.

One possible strategy to implement a productive infection is to silently infect respective host cells. It has been suggested that chlamydiae can benefit from a silent uptake resulting in increased survival and growth [15]. The most extensively studied example of a silent uptake into phagocytes is the clearance of apoptotic cells which is a well organized three step process [16]. First, apoptotic cells release “find-me” signals to recruit phagocytes to the site of apoptotic death [17]. Second, phagocytes recognize the presence of phosphatidylserine (PS) termed as “eat-me” signal on the membrane of apoptotic cells [16]. The final step is an active suppression of inflammation and immune response and can be termed as a “forget me” signal. This step is characterized by the release of anti-inflammatory cytokine TGF-ß and down-regulation of the pro-inflammatory cytokine TNF-α.

In this study we could show, that *C. pneumoniae* make use of these silent entry mechanisms to be taken up by monocyte- derived macrophages or alveolar macrophages via apoptotic PMN. Thus, *C. pneumoniae* efficiently infect and replicate inside PMN, which upon activation recruit monocytic phagocytes. *C. pneumoniae* infected PMN then become apoptotic as shown by PS and TUNEL positivity and are ingested by MF and AM, which secrete increased amounts of anti-inflammatory TGF-ß.

## Results

### *C. pneumoniae* remains transcriptionally active inside PMN

**Figure 1:**
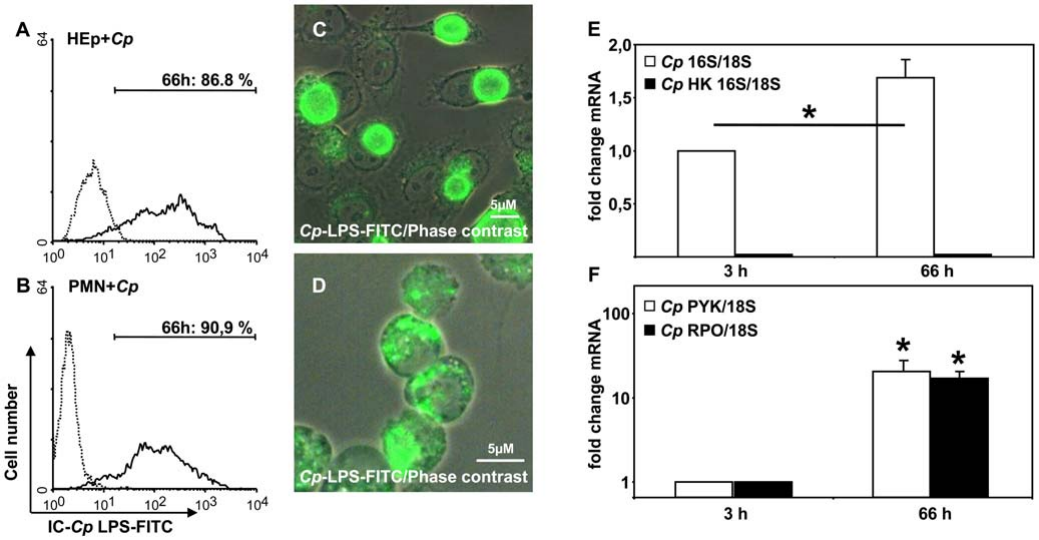
*C. pneumoniae* survives inside PMN. *C. pneumoniae* (*Cp*) infected HEp-2 cells and neutrophils (PMN) were analyzed 66 h p.i. for intracellular positivity of chlamydial LPS by FACS analysis (A, B; representative experiment out of 3) and fluorescence microscopy (C, D; magnification 630×). To analyze intracellular progeny of *C. pneumoniae* in PMN we performed real-time RT-PCRs of the 16S rRNA in comparison to host cell 18S rRNA using the ΔΔct- method for relative quantification. Viable (open bars) but not heat-killed (HK, closed bars) chlamydiae showed a significant increase in 16S rRNA expression (E) within 66 h p.i. (p = 0.02; n = 3). Enhanced transcriptional activity of *C. pneumoniae* inside PMN was proven by determining the expression of chlamydial genes pyk (open bars, p = 0.03) and rpoA (closed bars, p = 0.01) mRNA (F; n = 3).

To determine whether *C. pneumoniae* survives the uptake by PMN and remains viable intracellularly, we compared the infection with productive *C. pneumoniae* infection in HEp-2 cells. Using FACS we could show that 79%±2% (n = 3) of the HEp-2 cells and 83%±7% (n = 3) of the PMN stained *C. pneumoniae*- LPS positive 66 h p.i. (MOI 1), whereas non-infected cells alone stained negative (Fig. 1A,B). Microscopically, we observed that intracellular *C. pneumoniae* inclusions in PMN were morphologically different, showing multiple, smaller inclusions than in HEp-2 cells (Fig. 1C,D).

To prove intracellular viability of chlamydiae inside PMN we analyzed the transcriptional activity of chlamydial genes involved in pathogen replication and metabolism. Expression of the 16S rRNA of *C. pneumoniae* significantly increased from 3 h p.i. to 66 h p.i. when compared to the 18S rRNA expression of the host cells, indicating replication of chlamydiae inside PMN (Fig. 1E, p = 0.02). As a control, co- incubation of PMN with heat-killed (HK) chlamydiae did not result in detectable amounts of 16S rRNA (Fig. 1E). Key factors that indicate intracellular activity of chlamydiae like the RNA polymerase (rpo) and the pyruvate kinase (pyk) significantly increased within 66 h after PMN infection (Fig. 1F, p = 0.01 and p = 0.03, respectively).

### *C. pneumoniae* hides inside phosphatidylserine (PS)- positive apoptotic PMN

**Figure 2:**
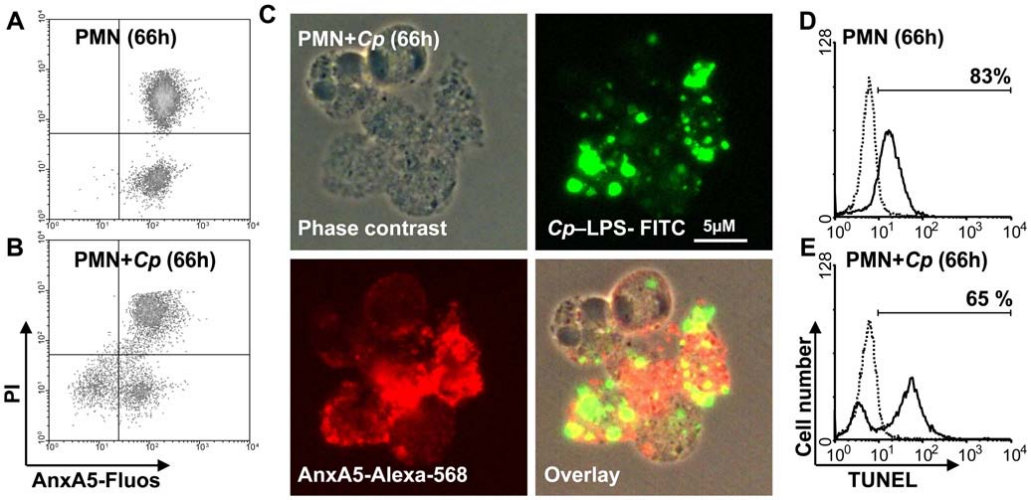
*C. pneumoniae* hides inside PS- positive PMN. To analyze early and late apoptotic markers, PMN were infected with *C. pneumoniae* or left in medium alone. Using Anxa5-fluos and propidium iodide (PI) staining we could show that both non- infected (>95%, A) and *C. pneumoniae*- infected (82%±12%, B) PMN become PS- positive within 66 h without significant changes in the amount of PI- positive necrotic cells (representative experiment out of 3). To visualize whether *C. pneumoniae*- infected PMN stain positive for PS we performed a double staining using a FITC- labeled anti-*C. pneumoniae* LPS mAb and Alexa568-labeled AnxA5 (C). In contrast, late apoptotic cells as determined by TUNEL staining (closed line) were more often found in the medium control than in *C. pneumoniae*- infected PMN (D, E; representative experiment out of 3).

PMN are short living cells. Even though *C. pneumoniae* infection delays apoptosis of PMN, 48%±8% of infected PMN become apoptotic 66 h p.i. [4]. Using Annexin A5 (AnxA5) staining to detect phosphatidylserine (PS) as an early marker of apoptosis on the outer cell membrane, we could detect that both non- infected (>95%, n = 3) and *C. pneumoniae*- infected (82%±12%, n = 3) PMN become PS- positive within 66 h (Fig. 2A,B). Additional staining with PI showed that *C. pneumoniae* infection does not increase the amount of necrotic cells compared to non- infected PMNs (Fig. 2A,B). To visualize whether *C. pneumoniae*- infected PMN stain positive for PS we performed a double staining using a FITC- labeled anti-*C. pneumoniae* LPS and Alexa568-labeled AnxA5 mAb (Fig. 2C). Within 66 h p.i. we observed that 86%±6% (n = 3) of *C. pneumoniae*- positive cells were expressing PS on the cell surface (Fig. 2C).

To analyze late apoptotic PMNs we further performed the TUNEL assay which reveals the apoptotic fragmentation of nuclear DNA. Whereas only 62%±3% (n = 3) of the *C. pneumoniae*- infected PMN became TUNEL- positive, more than 80% stained positive in the non- infected control 66 h p.i. (Fig. 2D,E).

### *C. pneumoniae* infected PMN release MIP-1ß, recruit and enter macrophages

**Figure 3:**
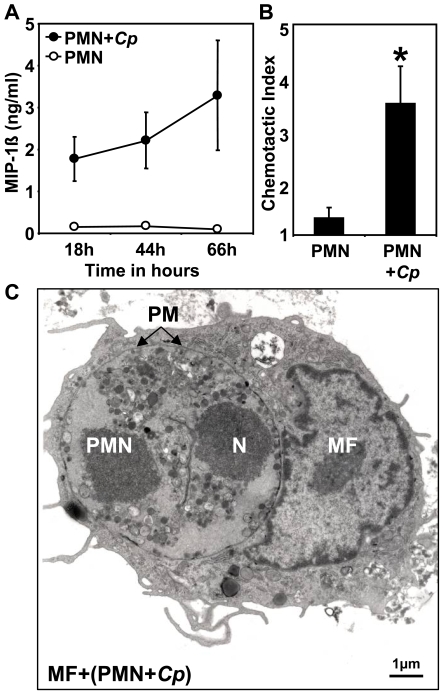
Increased MIP-1ß release in *C. pneumoniae* infected PMN. PMN were infected with *C. pneumoniae* (closed circles) or mock infected with HEp-2 lysates (open circles) (A). Increased secretion of MIP-1ß was observed in the supernatants of *C. pneumoniae*- infected PMN at the given time points by ELISA (A). The chemotactic index (specific migration/migration towards medium) indicates that monocytes are significantly better attracted by supernatants taken from *C. pneumoniae*- infected PMN than mock- infected control cells (B, p = 0.04; n = 3). Using transmission electron microscopy we could show that *C. pneumoniae*- infected PMN are engulfed by monocyte- derived macrophages (C). Arrows indicate the phagosomal membrane (PM) of the macrophage; containing a complete apoptotic PMN with condensed nucleus (N) (bar equals 1 µm, magnification 6000×).

Macrophages (MF) represent the first line of defense in the lung and are supposed to clear the lung from bacterial pathogens as well as apoptotic cells. First, we addressed the question whether MF are recruited by *C. pneumoniae*- infected PMN. A screening of chemotactic proteins revealed that *C. pneumoniae*- infection of PMN resulted in an increased production of MIP-1ß as compared to mock- stimulated PMN (Fig. 3A). Using an *in vitro* chemotaxis assay we found, that supernatants taken from *C. pneumoniae*- infected PMN attracted monocytes significantly better when compared to supernatants taken from mock- stimulated PMN (Fig. 3B, p = 0.04; n = 3). Having shown that PS- positive apoptotic PMN harbor viable chlamydiae and subsequently recruit monocytes, we wondered whether these cells are ingested by monocyte- derived macrophages. In a co- culture of PS- positive *C. pneumoniae*- infected PMN with MF, PMN were rapidly engulfed by MF (15 min) and could be observed within the MF phagosome by electron microscopy (Fig. 3C).

### PMN passage increases transmission of *C. pneumoniae* to macrophages

**Figure 4:**
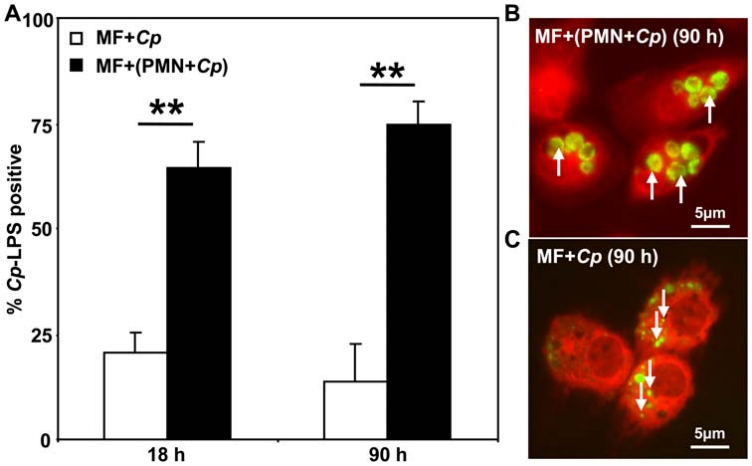
PMN passage increases chlamydial transmission to macrophages. *C. pneumoniae* alone (open bars) or *C. pneumoniae*- infected PMN (closed bars, 66 h p.i.) were co incubated with autologous macrophages (MF) at ratio of 1∶1 (A–C). The amount of *C. pneumoniae*- positive (*C. pneumoniae* LPS- staining) MF increased significantly after 18 and 90 h when MF were co incubated with *C. pneumoniae*- infected apoptotic PMN (A, p = 0.004), but not upon direct *C. pneumoniae* infection. Fluorescence microscopy revealed that co incubation of MF with *C. pneumoniae*- infected apoptotic PMN for 90 h resulted in multiple large inclusions (B) whereas directly infected MF showed smaller “persistent-like” inclusions (C; magnification 630×).

To follow the intracellular fate of *C. pneumoniae* in monocyte- derived macrophages (MF) and alveolar macrophages (AM) we used intracellular *C. pneumoniae* LPS- staining and FACS analysis. Whereas only 21%±5% (n = 3) of MF stained *C. pneumoniae* LPS- positive after direct *C. pneumoniae* infection (18 h p.i.), co- incubation of MF with *C. pneumoniae*- infected apoptotic PMN resulted in significantly higher amount of *C. pneumoniae* LPS- positive MF (65%±6%, p = 0.004; n = 3) (Fig. 4A). The higher efficacy in *C. pneumoniae-* infection of MF via apoptotic PMN was still observed 90 h p.i., showing more than 5-fold more *C. pneumoniae* LPS- positive cells compared to the direct infection of MF (75%±5% vs. 14%±8%, p = 0.004; n = 3). Increased bacterial load in MF that were infected through apoptotic PMN corresponded to larger intracellular *C. pneumoniae* inclusions (Fig. 4B) in comparison to directly infected MF (Fig. 4C).

**Figure 5:**
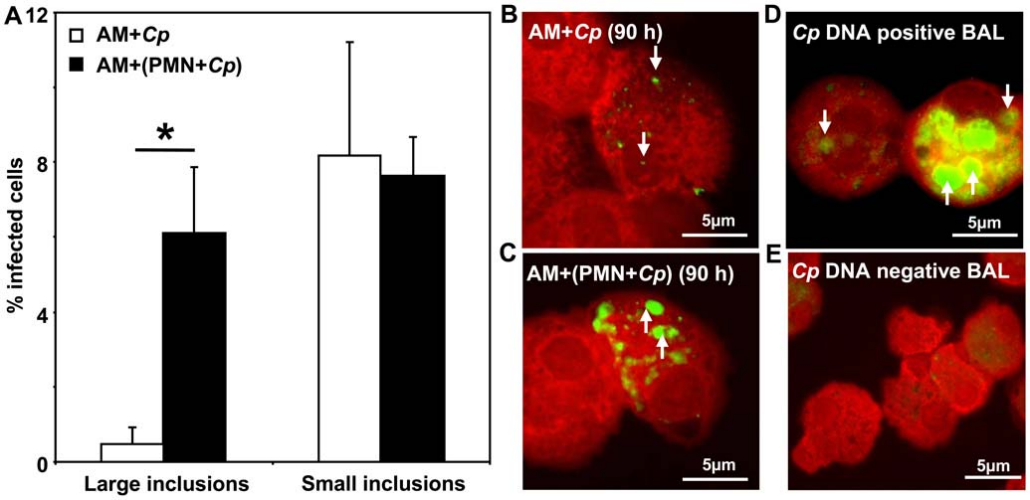
Alveolar macrophages harbor intracellular chlamydiae *in vivo*. Alveolar macrophages (AM) isolated from BAL fluids of *C. pneumoniae*- DNA negative patients were either directly infected with *C. pneumoniae* (open bars) or co incubated with *C. pneumoniae* infected PMN at a ratio of 1∶1 (closed bars, 66 h p.i.). The percentage of AM harvesting large and small inclusions were calculated 90 h p.i. by counting a minimum of 200 cells/slide using a FITC- labeled anti- *C. pneumoniae* LPS- staining protocol (A). Significantly more large chlamydial inclusions were detected in AM co incubated with *C. pneumoniae*- infected apoptotic PMN than in directly infected AM (A–C, p = 0.02). In addition, we analyzed AM from BAL fluids of patients suffering from community-acquired pneumonia (CAP) by fluorescence microscopy (D, E; representative experiment out of 4), showing both small, persistent-like inclusions (downward arrows) and large inclusions (upward arrows) in *C. pneumoniae*- DNA positive BAL (D) but not in *C. pneumoniae*- DNA negative BAL (E).

To bring the PMN passage strategy closer to respiratory *C. pneumoniae* infection, we repeated the experiments using purified alveolar macrophages (AM) from bronchoalveolar lavage (BAL) fluid. To analyze difference in the infection pattern of AM that were directly infected with *C. pneumoniae* vs. AM that were co- incubated with *C. pneumoniae*- infected apoptotic PMN we calculated the amount of small and large inclusions inside these cells (Fig. 5A–C). Thus, large inclusions were almost exclusively observed in AM that were infected by PS- positive *C. pneumoniae*- infected PMN (6%±1.8% vs. 0.5±0.5%, p = 0.02; n = 3), whereas the appearance of small inclusions did not differ between both conditions (Fig. 5A). The same pattern of intracellular chlamydial inclusions was observed in AM isolated from *C. pneumoniae*- DNA positive BAL fluids of patients with community-acquired pneumonia, but not in *C. pneumoniae*- DNA negative samples (Fig. 5D,E).

### Transmission of *C. pneumoniae* infection from PMN to macrophages depend on PS

**Figure 6:**
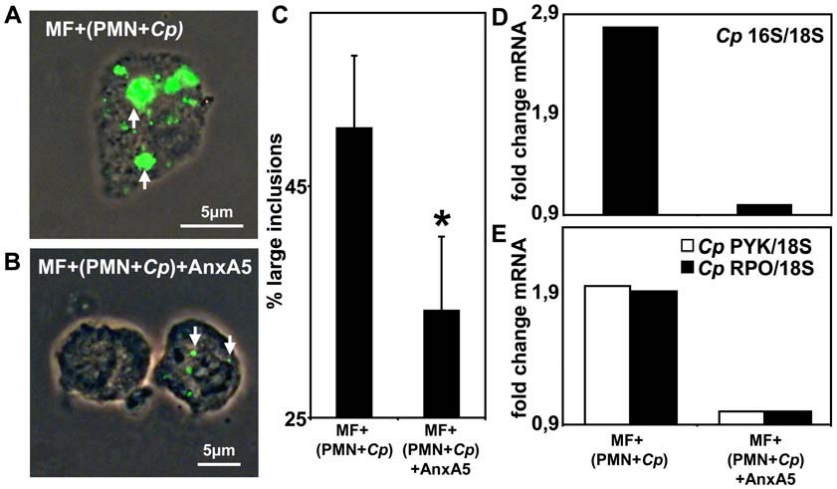
PS- dependent transmission of *C. pneumoniae* infection. Blocking of phosphatidylserine (PS) expression on *C. pneumoniae*- infected PMN by preincubation with recombinant AnxA5 significantly reduced the uptake of chlamydiae (A, B) and the formation of large inclusions (C, p = 0.02; n = 3) as shown by fluorescence microscopy with FITC- labeled anti- *C. pneumoniae* LPS- staining (representative experiment out of 3, magnification 630×) and calculation of a minimum of 200 cells/slide (C). Replicative activity of *C. pneumoniae*, indicated by the amount of 16S rRNA (D), pyk (open bars) and rpo (closed bars) mRNA expression compared to host cell 18S rRNA expression (E), decreased in MF when *C. pneumoniae*- infected PMN were preincubated with AnxA5 (representative experiment out of 3).

To investigate the mechanisms of *C. pneumoniae* transfer from apoptotic PMN to MF we tried to inhibit the uptake of apoptotic cells by blocking the apoptotic eat me signal PS on infected PMN. We compared MF that were co- incubated with *C. pneumoniae*- infected apoptotic PMN with MF that were co- incubated with *C. pneumoniae*- infected apoptotic PMN after preincubation with PS-binding AnxA5. Both types of MF stained positive for *C. pneumoniae*-LPS (Fig. 6A,B). However, the *C. pneumoniae* staining pattern in MF that were infected in the presence of PS- masking AnxA5 revealed less and smaller inclusions (Fig. 6B) similar to the staining we observed after direct infection of macrophages (Fig. 4C and Fig. 5B). We found that AnxA5 significantly reduced the number of large inclusions in MF, indicative for reduced bacterial load and replicative activity (Fig. 6C, p = 0.02). Using an AnxA5-based MACS separation system we were able to separate PS- positive *C. pneumoniae*- infected PMN from PS- negative cells, showing that only co- incubation with PS- positive PMN resulted in transfer of *C. pneumoniae* infection to MF (data not shown). Furthermore, expression of the chlamydial 16S rRNA in MF was decreased more than 2-fold when the PMN uptake was blocked by AnxA5 (Fig. 6D, n = 3). Reduced bacterial load in MF pre- incubated with AnxA5 was accompanied by reduced transcriptional activity of the rpo and pyk gene indicative for impaired growth and progeny of chlamydiae (Fig. 6E, n = 3).

### PS- dependent uptake of *C. pneumoniae* infected PMN silences MF immune response

**Figure 7:**
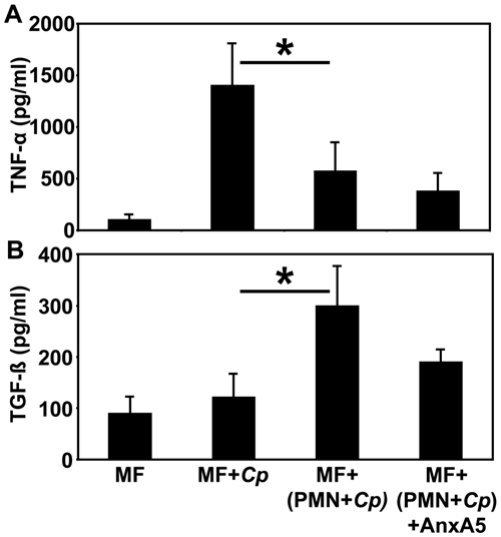
Silencing of MF immune response to chlamydial infection by PMN passage. Immune responses of directly *C. pneumoniae*- infected MF were analyzed in comparison to MF either co incubated with *C. pneumoniae*- infected PMN (66 h p.i.) or with *C. pneumoniae*- infected PMN after precinubation with recombinant AnxA5. Within 18 h p.i. supernatants of MF were collected to determine total amounts of TNF-α (A) and TGF-ß (B) by ELISA. Direct infection of MF with *C. pneumoniae* significantly increased TNF-α production (A, p = 0.01; n = 3), whereas the uptake of *C. pneumoniae*- infected apoptotic PMN was characterized by a significant up regulation of TGF-ß in MF (B, p = 0.02; n = 3).

We wondered whether the uptake of apoptotic *C. pneumoniae*- infected PMN would induce activation or silencing of MF, and therefore analyzed the expression of the pro- inflammatory and anti- inflammatory cytokines TNF-α and TGF-ß. We observed a significant reduction of TNF-α release from MF after uptake of *C. pneumoniae*- infected apoptotic PMN as compared to direct infection with *C. pneumoniae* (Fig. 7A, p = 0.01). In contrast, PMN that were not- infected by *C. pneumoniae* became secondarily necrotic after 3 days, and the uptake of these late apoptotic PMN resulted in enhanced TNF-α release when compared to non- stimulated macrophages alone (data not shown). In contrast, the uptake of *C. pneumoniae*- infected apoptotic PMN significantly increased the production of TGF-ß as compared to direct infection with *C. pneumoniae* (Fig. 7B, p = 0.02). Blocking PS on apoptotic PMN by AnxA5 pre- incubation reduced the production of TGF-ß but without reaching statistical significance (n = 3).

## Discussion

The life span of infectious *C. pneumoniae* elementary bodies (EB) is limited outside a human host cell. The pathogen strongly depends on the host cell environment for progeny and systemic dissemination. Alveolar macrophages (AM) are the pre-dominant cells in the human lung and represent the first line of defense in respiratory infections. Upon bacterial challenge the amount of PMN increases dramatically in the lung, accounting for more than 80% of the cells in acute respiratory tract infections. Whereas direct infection of macrophages results in clearance of the pathogen, uptake of *C. pneumoniae* by PMN has been shown to promote intracellular survival [4]. Nevertheless, although *C. pneumoniae* is frequently found inside PMN in early respiratory infections, the infection of macrophages seems crucial for systemic dissemination of the pathogen [1], [18]. *C. pneumoniae* survive and replicate inside PMN as shown by the transcriptional activity data and increased LPS expression, however, we did not succeed to transmit chlamydial infection from PMN to epithelial cells, which are primarily used for culture of chlamydiae. We found that in the presence of PMN lysates, containing high amounts of degrading enzymes from disrupted granules, chlamydial growth was precluded, even when epithelial cells were directly infected with *C. pneumoniae* (data not shown). Importantly, there is good evidence from *in vivo* data that the influx of PMN in acute lung infection with *C. pneumoniae* favors chlamydial growth and results in increased bacterial load in mice [19]. In contrast, lack of PMN recruitment in MyD88-deficient mice resulted in lower bacterial load of infected lungs, although the underlying mechanisms for enhanced chlamydial progeny and dissemination in the presence of PMN remained to be explained [19]. Gueinzius et al. could previously show, that PMN might serve as a vector for systemic *C. pneumoniae* dissemination as the infection can be transmitted to vascular endothelial cells [20]. However, mechanisms of cell to cell transfer of obligate intracellular bacteria are largely unknown, but it has been speculated that chlamydiae might profit from hiding inside apoptotic cells [15]. First evidence supporting this hypothesis came up recently as it has been demonstrated that *C. pneumoniae* can be transferred from UV-killed, PS-positive mouse embryonic fibroblasts to mouse DCs [21].

Blood monocytes and monocyte- derived macrophages are supposed to be the vector for systemic dissemination of *C. pneumoniae* throughout the human body. In up to 25% of healthy blood donors *C. pneumoniae* can be detected in circulating monocytes by PCR or culture [22]. Blood monocytes have been shown to harbor viable chlamydiae [23] which allow the transmission of the infection to endothelial or smooth muscle cells *in vitro* [24], [25]. Of clinical importance is the fact, that most of the chlamydiae enter a persistent-like state in monocytes that is refractory to current antibiotic treatment strategies [26], [27]. When compared to the infection of epithelial cells, growth of *C. pneumoniae* is strongly restricted in monocytes and monocyte- derived macrophages upon direct infection [24], [28].

The data presented here underline the importance for *C. pneumoniae* to be taken up by PMN to increase viability and virulence in macrophages. Sheltered by the “PMN envelope” and recognized by an apoptotic “eat-me signal” *C. pneumoniae* is ingested by MF and AM without being visible for the innate immune system.

The physiological clearance of tissue granulocytes is normally silent, but can under certain situations shift to become a pro-inflammatory event. For example, apoptotic cells that escape phagocytosis, disintegrate in a pro-inflammatory process called secondary necrosis. Importantly, uptake of non- infected late apoptotic PMN by MF did not result in “silencing” of the macrophage immune response. This is in line with findings from Afonso et al. showing decreased intracellular survival of Leishmania amazonensis when late apoptotic PMN were uptaken [29]. Recent studies demonstrated that also viruses and parasites misuse the apoptotic “eat-me signal” PS for productive infection [30], [31]. In general, phosphatidylserine (PS) on the outer membrane of apoptotic cells is the key element for induction of an anti-inflammatory environment. Therefore the mere presence of apoptotic cells at the site of infection is beneficial during the transfer of obligate intracellular pathogens from one to the next host cell. Blocking experiments with AnxA5 revealed that PS expression on apoptotic *C. pneumoniae* infected PMN is important for the productive transfer of not only *C. pneumoniae* but also *C. trachomatis* L2 (Fig. S1) from PMN into macrophages.

Regarding acute *C. pneumoniae* infections of the lower respiratory tract in humans, it seems to be a footrace between direct clearance of *C. pneumoniae* by AM and ingestion of *C. pneumoniae* by PMN which might increase chlamydial pathogenicity. Whether direct clearance of *C. pneumoniae* by MF results in complete eradication of the pathogen or promotes persistence of chlamydiae in the lung still has to be determined. Morphological analysis of *C. pneumoniae* inclusions in AM from *C. pneumoniae*- DNA positive BAL fluids suggests that direct and indirect ingestion of chlamydiae may occur *in vivo*.

These data show that *C. pneumoniae* infection of MF results in increased pathogen activity and silenced immune response when transferred by apoptotic PMN. However in acute infection, the innate and acquired immune mechanisms will be turned on by direct host-pathogen interactions in order to limit infection to the lungs. Thus, pro- inflammatory immune responses will be generated by direct contact of chlamydial TLR- ligands with its specific receptors [19], [32] and MHC class I-restricted lysis of infected cells by CD8(+) CTL will occur to prevent systemic dissemination of the pathogen [33].

We demonstrated that *C. pneumoniae* remain transcriptionally active inside PMN and induce the release of macrophage attracting MIP-1ß. *C. pneumoniae* hiding inside apoptotic PMN were phagocytosed by MF and developed multiple large inclusions inside MF. In contrast, direct *C. pneumoniae* infection of MF resulted in a persistent-like infection. Efficient uptake and intracellular development of *C. pneumoniae* in MF via apoptotic PMN was dependent on the expression of phosphatidylserine on infected cells. Our data suggest that *C. pneumoniae* misuses central pathways of apoptotic cell clearance to survive inside human cells. Further studies are needed to elucidate the functional role of this mechanism in the dissemination of chlamydiae from the lung to the circulation in humans after acute respiratory infection.

## Materials and Methods

### Bacterial strain, cell preparation and infection experiments

The *C. pneumoniae* strain CV-6, used in this study, was isolated from a coronary artery plaque and continuously propagated on HEp-2 cells as described [34].

PMN and monocyte- derived macrophages (MF) were generated from buffy coat blood as described previously [4], [35]. Alveolar macrophages (AM) were isolated from bronchoalveolar lavage (BAL) fluid [8] from healthy volunteers (*C. pneumoniae*- DNA negative) and patients with acute *C. pneumoniae* infection as proven by *C. pneumoniae*- DNA positive PCR results from BAL (n = 4). Cells were cultured for 1 to 4 days at 37°C in a humidified atmosphere (5% CO_2_) in RPMI 1640 medium, containing 50 µM 2-mercaptoethanol, 2 mM L-glutamine, 10 mM HEPES complemented with 10% FCS (all Sigma-Aldrich, Munich, Germany).

PMN (1×10^7^/ml) were co- incubated with *C. pneumoniae* at a ratio of 1∶1 (MOI 1), equivalent amounts of heat-killed *C. pneumoniae*, or with mock infected HEp-2 cell lysates as negative controls. 3 hours after co incubation PMN were separated from extracellular *C. pneumoniae* by several washing and centrifugation steps at 200×g. MF or AM were co- incubated with *C. pneumoniae*- infected PMN (66 h p.i.) at a PMN to macrophage ratio of 1∶1, or directly infected with *C. pneumoniae* with a MOI 1. Blocking experiments for phosphatidylserine specific uptake of *C. pneumoniae*- infected PMN were performed with recombinant Annexin A5 (AnxA5; Responsif GmbH, Erlangen Germany) at a concentration of 5 µg/1×10^6^ PMN.

### Immunohistochemistry, Western blot analysis and electron microscopy of infected cells

For *C. pneumoniae*- specific staining the cells were cytocentrifuged, fixed in methanol and stained using a FITC-conjugated anti-*C. pneumoniae* mAb (clone RR402, IgG3, Dako, Hamburg, Germany) or a FITC-conjugated isotype matched control mAb (Dako), followed by counterstaining with Evans blue. Inclusion morphology was analyzed under a Zeiss Axioskop-2® fluorescent microscope fitted with HRS- Axiocam® and Axiovision software 4.5®. Percentages of *C. pneumoniae*- LPS positivity was calculated by counting a minimum of 200 cells/slide. For structure preservation electron microscopy, MF were co- incubated for 15 min with *C. pneumoniae* - infected PMN at a ratio of 1∶1. Cells were fixed and examined with a Philips EM 400 electron microscope as described [35].

### Flow cytometry analysis

Intracellular *C. pneumoniae* LPS was quantified using FACS analysis. Cells were permeabilized using the Cytofix/Cytoperm Plus Kit (BD Biosciences, Heidelberg, Germany) as recommended by the manufacturer and stained with a FITC-conjugated anti- *C. pneumoniae* mAb or an isotype matched control (both Dako). Annexin A5 (AnxA5), propidium iodide and TUNEL staining were performed as described [4]. In addition we stained cells with Alexa568-conjugated AnxA5 followed by an intracellular *C. pneumoniae* LPS staining as described above. To maintain AnxA5 binding to PS the intracellular *C. pneumoniae* staining was performed in the presence of 5 mM CaCl_2_.

### Real-Time PCR

Total RNA isolation, generation of cDNA and PCR amplification was performed as described [36]. The expression of the chlamydial 16S rRNA (forward [TCG CCT GGG AAT AAG AGA GA]; reverse [AAT GCT GAC TTG GGG TTG AG]), rpoA (forward [GCAATCGAAGGGGTTATTGA]; reverse [TGATCTGGGTTAACG GCTTC]), pyk (forward [AGC TTG CGG ATG GAA TTA TG]; reverse [ATG CAG TTT CCC CTG ACA AC]), and 18S rRNA (forward [TCA AGA ACG AAA GTC GGA GG], reverse [GGA CAT CTA AGG GCA TCA CA]) was analyzed by relative quantification using the ΔΔct- method as shown before [36]. Changes in the mRNA expression profile over time were calculated by comparing values for 66 h p.i. to values for 3 h p.i. which were set to 1 for clear presentation.

### Cytokine measurement and chemotaxis assay

Cells were cultured and supernatants were collected after given time points and stored at -20°C until cytokine determination. MIP-1ß, TNF-α and TGF-ß was measured using ELISA (R&D Systems, Wiesbaden, Germany) according to the manufacturer's instructions (duplicate assays for at least 3 independent experiments). Chemotaxis assays were performed with freshly isolated monocytes in 24-well Transwell plates (Costar, Bodenheim, Germany) as described before [35]. The chemotactic index (CI) was calculated by dividing the number of migrated cells towards supernatants taken from *C. pneumoniae*- infected PMN, divided by the number of cells migrated in medium alone.

### Statistical analysis

Data are depicted as mean±standard error of the mean. Statistical significance of the results was analyzed with Student's t test and Microsoft Excel 8.0® software. Results were considered statistically significant at *p*<0.05 (^*^) and *p*<0.005 (^**^).

## Supporting Information

Figure S1 PS- dependent transmission of *C. trachomatis* infection. Blocking of phosphatidylserine (PS) expression on *C. trachomatis (L2)* infected PMN by preincubation with recombinant AnxA5 significantly reduced the uptake of chlamydiae (n = 4, p = 0.005). Percentages of *C. trachomatis* - LPS positivity was calculated by counting a minimum of 200 cells/slide stained with FITC- labeled anti- chlamydial - LPS staining.
(0.64 MB TIF)
