# Supplementary figures and images for: Chlamydia pneumoniae Hides inside Apoptotic Neutrophils to Silently Infect and Propagate in Macrophages
Source: PLoS One. 2009 Jun 23;4(6):e6020. doi: 10.1371/journal.pone.0006020 (PMC2695784; doi:10.1371/journal.pone.0006020)

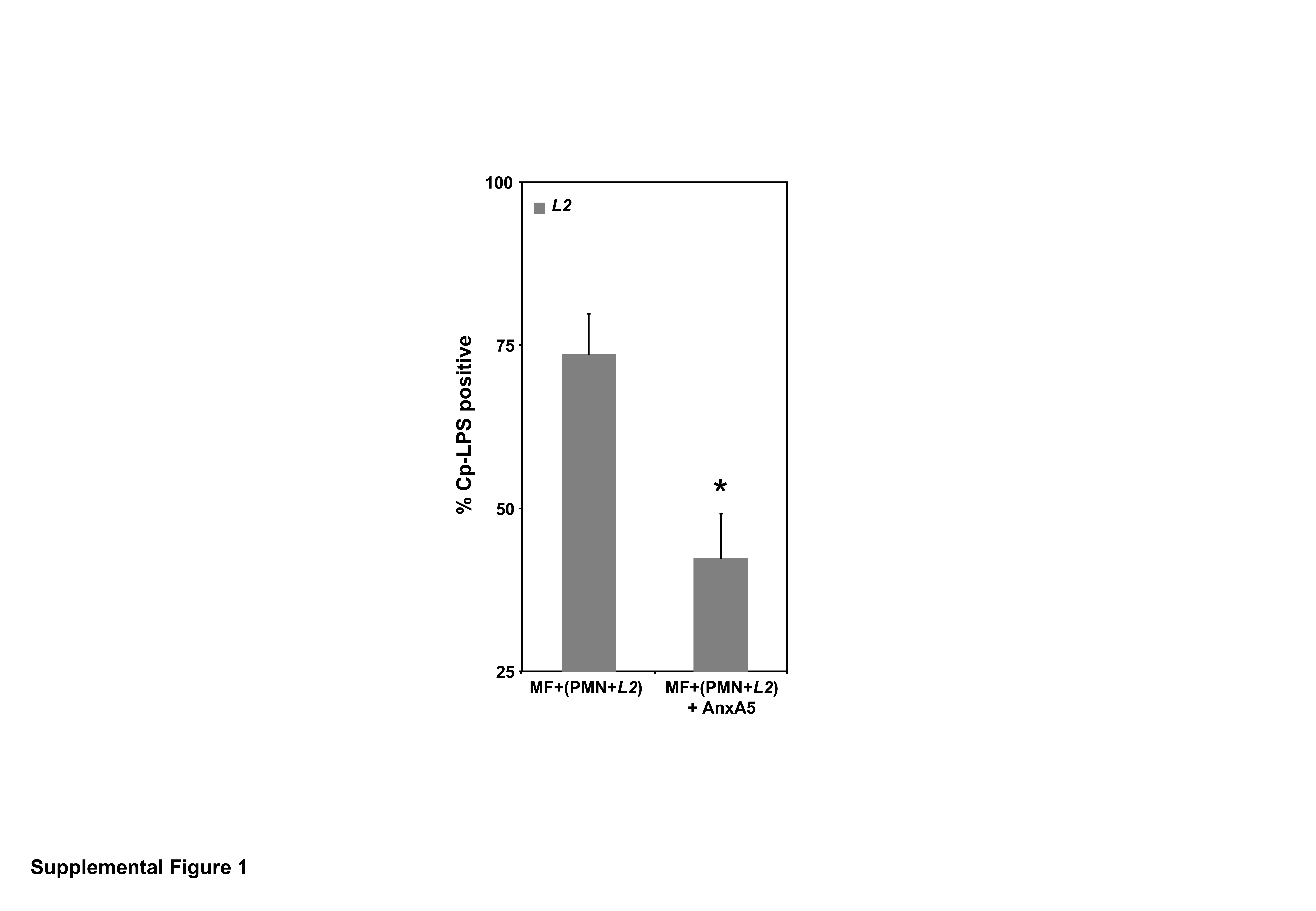

Supplement: Figure S1 — PS- dependent transmission of C. trachomatis infection. Blocking of phosphatidylserine (PS) expression on C. trachomatis (L2) infected PMN by preincubation with recombinant AnxA5 significantly reduced the uptake of chlamydiae (n = 4, p = 0.005). Percentages of C. trachomatis - LPS positivity was calculated by counting a minimum of 200 cells/slide stained with FITC- labeled anti- chlamydial - LPS staining. (0.64 MB TIF) [file pone.0006020.s001.tif]
